# Early childhood caries risk indicators among preschool children in rural Egypt: a case control study

**DOI:** 10.1186/s12903-023-03771-9

**Published:** 2024-01-03

**Authors:** Dina Attia, Mona K. ElKashlan, Susan M. Saleh

**Affiliations:** Department of Pediatric Dentistry and Dental Public Health, Faculty of Dentistry, Alexandria University, Alexandria, Egypt

**Keywords:** ECC, Socioeconomic status, Oral hygiene, Dmf, Saliva, Egypt

## Abstract

**Background:**

Early childhood caries (ECC) is a public health problem, especially in developing countries like Egypt which has an ECC prevalence of 74%. This research aimed to assess the risk indicators associated with ECC in a rural, socially-disadvantaged population in Alexandria, Egypt.

**Methods:**

A case-control study was conducted in 8 nurseries of preschool children aged 3 to 5 years in rural, deprived areas in Alexandria, Egypt, from October 2019 till January 2020. Two groups, 93 with ECC and 93 without ECC. A validated questionnaire was used to collect sociodemographic data including age, sex, number of siblings, socioeconomic status, oral health practices including toothbrushing frequency, pattern of dental visits, daily frequency of sugary snacks. Also, salivary pH and buffering capacity were assessed. A trained and calibrated dentist assessed caries status clinically according to the World Health Organization (WHO) criteria using the dmft index and oral hygiene status using Silness and Loe Plaque Index. Chi-squared test, followed by multivariable logistic regression were performed to assess the relation between independent variables and ECC, *P* < 0.05 was considered to be statistically significant.

**Results:**

Bivariate analysis showed that age, mother’s education, dental visits, dietary habits, Plaque index, salivary pH and buffering capacity were significantly associated with ECC. The significant risk indicators for ECC in multivariable regression were age (AOR = 4.73, 95% CI: 2.76–7.83), mother’s education (illiterate vs. university educated, AOR = 28.36, 95% CI: 8.51-112.92), frequency of daily sugary snacks (twice vs. once, AOR = 2.00, 95% CI: 1.29–3.49, and three or more vs. once, AOR = 2.67, 95% CI: 1.72–3.27), night feeding (AOR = 1.89, 95% CI: 1.38–10.21), Plaque index (AOR = 21.34, 95% CI: 5.56–81.99), and salivary pH (AOR = 0.16, 95% CI: 0.05–0.58).

**Conclusion:**

This study suggests that sociodemographic indicators, dietary habits, plaque accumulation and salivary pH are risk indicators for ECC in the studied population.

## Introduction

ECC is defined by The American Academy of Pediatric Dentistry (AAPD) as the presence of one or more decayed (non-cavitated or cavitated lesion), missing (due to caries), or filled tooth surfaces in any primary tooth in a child 71 months of age or younger [1]. ECC is still a critical public health problem in both developing and developed countries. According to the Global Burden of Disease Study 2017, 532 million children suffered from untreated dental caries in their deciduous teeth [2]. As for Egypt, a high prevalence of ECC 74% was reported in the last survey carried out in 2018 [3].

It is a multifactorial disease that takes place due to the interaction of several factors including susceptible tooth surface, fermentable carbohydrates, and cariogenic microorganisms across time. Numerous risk indicators are associated with ECC onset and progression, such as sociodemographic factors including age, parents’ education level and occupation, biological factors, such as salivary pH, buffering capacity, flow rate, dietary factors, such as breast/bottle feeding, night feeding, frequency of sugary snacks, and oral-health related behaviors, such as toothbrushing frequency, dental visits [4, 5].

The serious consequences of untreated ECC include pain, infection, chewing difficulty, malnutrition, gastrointestinal disorders, and low self-esteem [6]. Furthermore, preschoolers with ECC have a higher risk for the development of new caries lesions in the future. Children from disadvantaged families are mostly affected by more severe ECC. In addition, several cross-sectional studies have consistently shown that individuals with higher socioeconomic status (SES) generally adopt healthier practices and experience better oral health outcomes compared to those with lower SES [7, 8].

To effectively manage ECC, it is crucial to adopt prevention approaches that consider the individual’s caries risk. By implementing such risk-based strategies, the control of ECC can be improved [9]. Therefore, it is essential to investigate the risk factors associated with ECC, in order to control the disease and promote better oral health among children. Egypt population has exceeded 100 million inhabitants; among this population is more than 15 million individuals who are younger than 6 years old, and 57% of whom live in rural areas [10]. However, few studies in Egypt have assessed the risk factors of ECC in rural, socially disadvantaged areas. Hence, the aim of the current study was to assess the risk indicators associated with ECC in rural areas in Alexandria, Egypt.

## Materials and methods

### Study design and setting

This case control study was carried out between October 2019 and January 2020 in rural areas in Alexandria, Egypt. We had administrative access to 5 villages which had 39 nurseries. Multistage random sampling was used in the current study, where in the first stage 8 nurseries were selected randomly in proportion to the number of people in these villages. In the second stage, children were chosen from the nurseries in a stratified sampling strategy according to the sex proportions. Children were divided into 2 groups, children with 1 or more carious teeth; and caries-free children.

Ethical approval was obtained by Dental Research Ethics Committee Faculty of Dentistry, Alexandria University, prior to commencement of study. Parents/caregivers of all children were asked to provide a written informed consent for joining the study after thorough explanation of the study aims and procedure. In addition, approval from the nurseries management was obtained after explaining the study.

### Participants

Eligibility criteria included healthy children aged 3 to 5 years old, without any systemic diseases, who were cooperative with the examiner. Children who used antibiotics and topical fluoride application one month prior to the study were excluded from the study. Children who exhibited uncooperative behavior or cried during saliva sampling were excluded from the study. Sample size was calculated using Epitools Epidemiological Calculators, Ausvet. (Available at: https://epitools.ausvet.com.au/casecontrolss) with a 95% confidence interval, 5% standard error, 0.74 probability of exposure in controls [3], and expected OR 2.78 for those who snacked more than thrice per day [11]. The total required sample size was 208, 104 cases and 104 controls.

### Study measures

Data were collected using a questionnaire, oral examination, and saliva sampling. Outcome variable in the current study was ECC, exposure variables were dietary habits; and potential confounders were sociodemographic profile and oral health related behaviors.

### Questionnaire

The questionnaire was developed based on the studies by Mahesh et al. and Özen et al. [11, 12]. The questionnaire included 3 sections. Section 1 assessed the sociodemographic profile of the child including age, sex, number of siblings; mother’s education (illiterate, secondary school or less, university and above); and father’s employment (employed or not). Section 2 assessed the oral-health related behaviors including pattern of dental visits (regular, on pain, never) and toothbrushing frequency (brushed once daily or no). Section 3 assessed dietary habits by 2 questions, asking if the child was fed at night by breast or bottle (yes/no), and the frequency of sugary snacks (once, twice, or three times or more per day).

The questionnaire was initially developed in English, then translated to Arabic, then back translated to English to ensure accuracy. Content validity was assessed by 3 experts and the Arabic version was pilot tested on 20 parents to make sure that it was clearly understandable. The data of the pilot test was not included in the final analysis. Parents/caregivers were approached in the early morning when they dropped off their children to the nursery. Interview-based questionnaire was used to collect data.

### Oral examination

Dental examination was carried out by one trained and calibrated dentist (DA). Intra-examiner agreement was assessed by re-examination of 10% of the sample for caries, at a 7-day interval with Kappa = 0.89, indicating excellent intra-examiner reproducibility. All children were examined under sunlight near to the window in the class, while sitting on regular chair and using flat disposable dental mirrors and blunt tip dental probes. Oral hygiene status was assessed using the Silness and Loe Plaque index to assess the thickness of plaque at the gingival area of 6 index teeth [13]. The World Health Organization criteria for caries diagnosis were followed to evaluate the caries status for all children. Soft debris covering the teeth was removed by gauze, followed by visual examination and probing to detect caries at cavitation level [14]. Teeth exhibiting white spot lesions were considered sound, teeth with temporary filling or restorations showing signs of decay were considered decayed. No radiographic examination was done. All erupted teeth were assessed and coded as d (cavitated), f (filled) or m (missed due to caries).

### Salivary sampling

Saliva samples were collected from children in the morning between 9 and 11 am, and after a fasting period of two hours as instructed to caregiver at time of questionnaire collection to standardize the time of collection. All children were asked to swallow the pre-existing saliva to clear the mouth of any residual saliva. Unstimulated whole saliva was collected with the children’s heads tilted slightly forward. The children were asked not to swallow or move the tongue or lips during the collection period. Saliva was allowed to drip off the lower lip into a graduated test tube until 3 ml were obtained for biochemical analysis [15]. The collected saliva was transferred into sterile plastic centrifuging tubes, placed on dry ice and transported within 1 to 2 h to the Biochemistry Laboratory in the Faculty of Medicine, Alexandria University. The pH and buffering capacity were measured instantly in the biochemistry lab. As for the initial pH, it was measured using a digital portable pH meter (Adwa AD 111, Hungary). The pH meter was calibrated before the start then immersed into a test tube containing 2 ml saliva, and the reading was allowed to stabilize for a few seconds before being recorded. The buffering capacity was determined by titration of 0.1 ml of 0.01 N HCl solution added to 1 ml saliva, using an automatic micropipette (BioSTC pipette, Germany). After each addition of the acid, the change in the pH was monitored using a portable pH meter. This process was repeated until reaching pH 4.0; the buffering capacity of saliva was expressed as the total volume in ml of acid added to one ml of saliva to change its initial pH value to pH of 4 [16].

### Statistical analysis

Statistical analysis was carried out using statistical package for social sciences (SPSS for Mac OS X, version 23.0, Inc. Chicago, IL, USA). The study groups were compared regarding sociodemographic factors, oral health behaviors, and dietary habits using Chi square test. The mean values of the quantitative variables like caries experience, Silness and Loe Plaque index, buffering capacity and pH were compared using student-t test. Multivariable logistic regression analysis was used to assess the relation between the indicators and ECC status (present/ absent). *P* < 0.05 was considered to be statistically significant.

## Results

A total of 186 preschool children, with an age ranging between 3 and 5 years, were recruited to the study. The mean ± SD age of the children was 4.1 ± 0.8 years and 52.7% were females. Table 1 displays the differences in risk indicators between the study groups. There were no significant differences between groups in child sex (*p* = 0.87), number of siblings (*p* = 0.74), father’s employment (*p* = 0.85), and use of toothbrush (*p* = 0.40). Children with ECC were significantly older than children without ECC (*p* < 0.001), had less educated mothers (*p* = 0.001), were more likely to visit the dentist on pain (*p* < 0.001), more fed at night (*p* = 0.001), and consumed more sugary snacks daily (*p* < 0.001). In addition, ECC children had significantly higher scores of Silness and Loe Plaque index (*p* < 0.001), lower values of salivary pH (*p* < 0.001) and buffering capacity (*p* = 0.04) than children without ECC.

**Table 1:** Sociodemographic background, oral health-related behaviors, and dietary habits in children with and without ECC.

	Children with ECC n= (93)		Children without ECC n= (93)	*P* value
Sociodemographic profile				
Sex				
Male: n (%) Female: n (%)	43 (46.2) 50 (53.8)		45 (48.4) 48 (51.6)	0.87
Age				
Mean ± SD	4.5 ± 0.6		3.8 ± 0.7	< 0.001^*^
Number of siblings				
Mean ± SD	1.69 ± 0.96		1.64 ± 1.02	0.74
Mother’s education				
Illiterate: n (%) Secondary or less: n (%) College completed: n (%)	15 (16.1) 59 (63.4) 19 (20.5)		1 (1.1) 58 (62.4) 34 (36.6)	0.001^*^
Father’s employment				
Employed Unemployed	83 (89.2) 10 (10.8)		86 (92.5) 7 (7.5)	0.85
Oral health-related practices				
Pattern of dental visits				
Never: n (%) Regular: n (%) On pain: n (%)	24 (25.8) 7 (7.5) 62 (66.7)	71 (76.3) 16 (17.2) 6 (6.5)		< 0.001^*^
Use of toothbrush				
Yes: n (%) No: n (%)	64 (68.9) 29 (31.1)	69 (74.2) 24 (25.8)		0.81
Daily use of toothbrush				
Yes: n (%) No: n (%)	20 (21.5) 73 (78.5)	28 (30.0) 65 (70.0)		0.40
Dietary habits				
Night feeding				
Yes: n (%) No: n (%)	70 (75.3) 23 (24.7)	29 (31.2) 64 (68.8)		0.001*
Frequency of sugary snacking				
Once: n (%) Twice: n (%) Three or more: n (%)	17 (18.3) 32 (34.4) 44 (47.3)	49 (52.7) 28 (30.1) 16 (17.2)		< 0.001^*^
Oral hygiene status				
Plaque index				
Mean ± SD	0.78 ± 0.33	0.54 ± 0.23		< 0.001^*^
Salivary parameters				
Salivary pH				
Mean ± SD	7.46 ± 0.41	7.71 ± 0.47		0.001^*^
Buffering capacity				
Mean ± SD	0.91 ± 0.18	0.97 ± 0.14		0.04*

* Statistically significant at *P* < 0.05

**Fig. 1:**
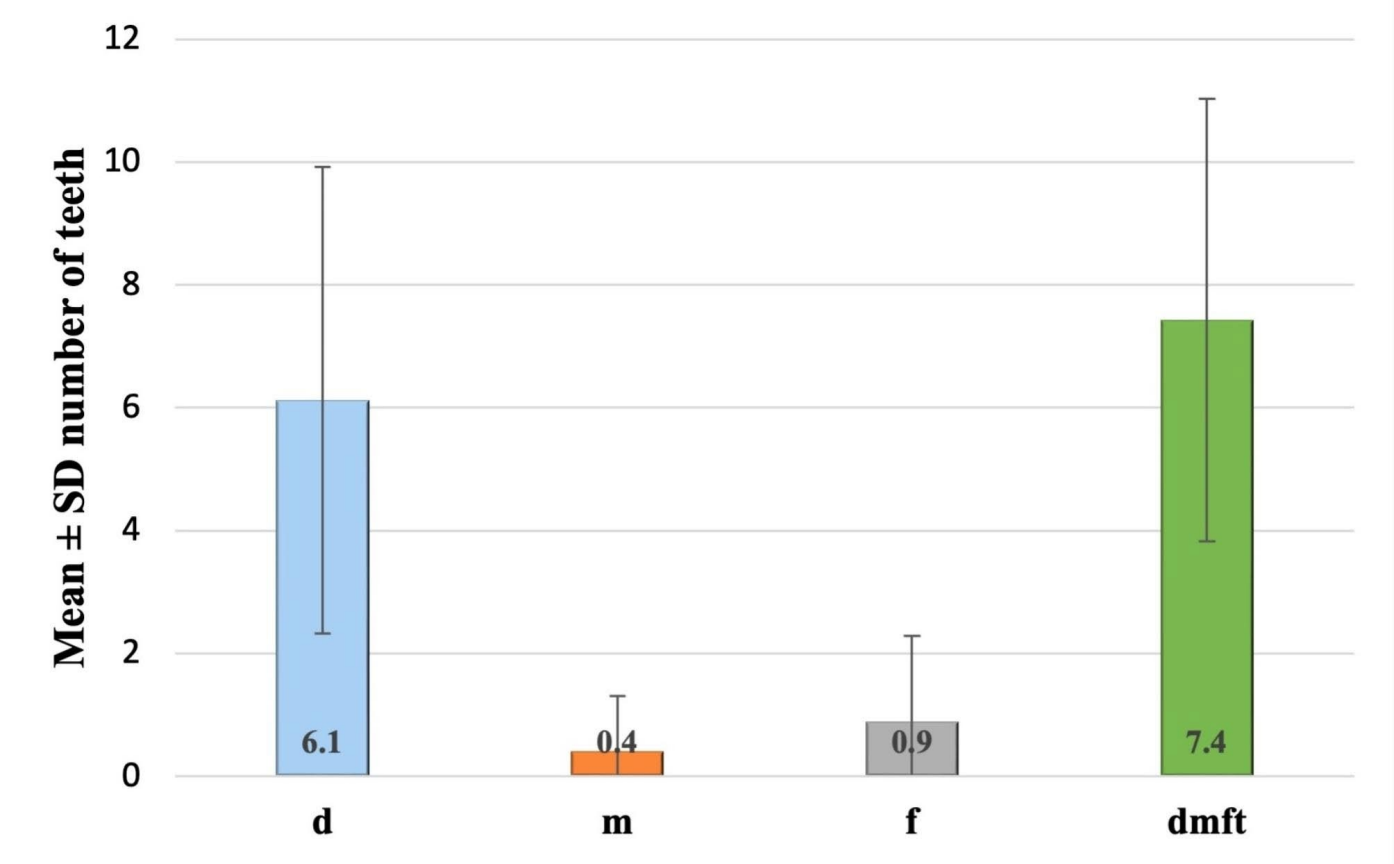
Caries experience of children with ECC.

The total dmft in children with ECC ranged from 2 to 20 teeth with a mean ± SD of 7.43 ± 3.63, and the highest component was decayed teeth (d), mean ± SD = 6.12 ± 3.8. (Fig. 1)

Table 2 shows regression analysis for factors associated with ECC. The risk indicators significantly associated with higher odds of ECC were child’s age (AOR = 4.73, 95% CI: 2.76–7.83), having illiterate mothers (AOR = 28.36, 95% CI: 8.51-112.92), frequency of daily sugary snacks (twice per day compared to once per day AOR = 2.00, 95%CI 1.29–3.94, and three times or more compared to once per day, AOR = 2.67, 95% CI 1.72–3.27), night feeding (AOR = 1.89, 95% CI 1.38–10.21) and Plaque index (AOR = 21.34, 95% CI 5.56–81.99). Salivary pH was associated with significantly lower odds of ECC (AOR = 0.16, 95% CI 0.05–0.58).

**Table 2:** Association between socio-demographic factors, oral health behaviors, dietary habits, oral hygiene, salivary parameters and ECC status in multivariable logistic regression

Variables		AOR	95% C.I.	*P* Value
Age		4.73	2.76, 7.83	< 0.001^*^
Mother’s education	Illiterate vs. university educated	28.36	8.51, 112.92	0.003^*^
	Secondary vs. university educated	2.71	0.92, 7.99	0.07
Pattern of dental visit	On pain vs. regular	1.12	0.83, 33.17	0.32
	Never vs. regular	1.48	0.95, 24.32	0.09
Frequency of daily sugary snacks	Twice vs. once	2.00	1.29, 3.94	< 0.001^*^
	Three or more vs. once	2.67	1.72, 3.27	0.006^*^
Night feeding	Yes vs. No	1.89	1.38, 10.21	0.01*
Plaque index		21.34	5.56, 81.99	< 0.001^*^
Salivary pH		0.16	0.05, 0.58	0.002^*^
Buffering capacity		1.21	0.07, 20.92	0.89

AOR: adjusted odds ratio

C.I.: confidence interval

*: statistically significant at *P* < 0.05

## Discussion

This case control study aimed to identify factors that may be related to ECC in rural, socially disadvantaged areas in Alexandria, Egypt, and that may become the focus for interventions aimed at the prevention of ECC. The study revealed that age, mother’s education, frequency of daily sugary snacks, night feeding, and oral health status can be considered as potential risk factors for ECC in rural areas.

The study showed that decayed teeth constituted the majority of teeth with caries experience which agrees with previous studies conducted in Egypt in different settings [17, 18]. This finding may be explained by limited access to care, especially considering the low number of pediatric dentists in Egypt [19], and that preschool children are not covered by dental health insurance like schoolchildren [20]. Another reason may be related to the limited awareness of the importance of primary dentition for the general wellbeing of the child and normal eruption of the permanent dentition [21].

In the current study, age was significantly associated with ECC, where older children had higher odds of having ECC. This agrees with several previous research [3, 22, 23], which could be attributed to the increasing number of erupted primary teeth, and teeth becoming more exposed to the oral environment and various cariogenic challenges. Study findings revealed that mother’s education was significantly associated with ECC, and father’s employment status was not associated with the presence of ECC. This could be because mothers tend to spend more time with their children in this rural community than fathers, and may, thus, have a greater role in shaping their children’s oral hygiene practices and dietary habits [24, 25]. The educational background of mothers might influence their knowledge and understanding of the importance of oral health, leading to more effective oral care practices and better dietary choices for their children. Several authors have discussed the association between parental socioeconomic status and ECC in their children and pointed to higher odds of ECC in children whose parents had low education level [22, 26].

The study investigated a number of oral health habits. Among them, toothbrushing was not significantly associated with the presence of ECC, this finding agrees with Mallineni et al. [23]. This may be due to the fact that preschool children may lack the manual dexterity required to remove dental plaque and maintain good oral hygiene, therefore, no difference was found regarding ECC between children who brushed their teeth and those who did not. Thus, it is important to educate parents about teaching and assisting their children in brushing their teeth in a correct manner to reduce caries risk, and develop the habit of effective brushing in their children at a young age. The study also showed that the reasons of dental visits were not significantly associated with ECC. This is in accordance with Cianetti et al. [26] who reported no difference in caries presence between the children who visited at least once the dentist and children who had never visited the dentist. The main reason in the current study for dental visits was predominantly problem/treatment-related; such a finding points to the wrong concept adopted by many people that dental visits are only needed when there is pain, which again points to the lack of awareness of the importance of periodic dental check-ups for prevention as well as early and prompt treatment.

The study findings showed a significant association between ECC presence and night feeding and the frequency of daily sugary intake. This is consistent with findings reported by Jain et al. [27], and Kabil and Eltawil [28] and emphasizes the prominent role of sugars in this population where only a minority of children brush their teeth daily and in a country which has the highest position of sugar consumption in Africa [29]. Night feeding may also have a detrimental effect on oral health due to decreased salivary flow during sleep which reduces the clearance of liquid carbohydrates from the oral cavity, creating an environment conducive to the growth of cariogenic microorganisms and promoting caries. The finding is important considering the high prevalence of sugar associated diseases, such as caries, diabetes mellitus, and cardiovascular diseases among Egyptians [30]. Sugar consumption may thus be a worthy common risk factor to address, and promoting healthy dietary habits at an early age may have high return on investments throughout life. Upstream, policy measures such as sugar taxation may be able to support behavior change modification interventions.

Finally, The study showed significantly lower values of salivary pH and buffering capacity among children with ECC which agrees with Pyati et al. [31]. However, in multivariable analysis, only salivary pH was significantly associated with ECC. This could be attributed to the notion that saliva buffering capacity works more efficiently during stimulated flow rates and the saliva collected in the current study was unstimulated [32].

Based on the findings of this study, it can be assumed that, categorizing children according to their socioeconomic status as evident by their parents’ education and occupation, in addition to monitoring their oral health using oral hygiene indices would be an accurate and economic method to identify children who are at risk for ECC in rural, socially disadvantaged populations. This approach is deemed financially viable for identifying tooth decay in countries with limited economic resources, such as Egypt, that falls within the low- and middle-income range. If feasible, it is also possible to combine this with the assessment of pH levels in saliva. In addition, oral health programs stressing on the parental role in improving oral health of young children should be implemented.

The study had some limitations. First, there is some potential for recall bias similar to all case control studies [33]. Because it occurs in both cases and controls, it would unlikely affect the conclusions. Second, the number of included children is relatively small because data collection had to be stopped due to the COVID-19 pandemic and restricted physical mobility. Despite this small sample size, the study was adequately powered to detect important associations. Third, we did not explicitly assess the use of fluoridated toothpaste although the question about the frequency of toothbrushing implicitly gives an indication since almost all toothpaste brands in the Egyptian market are fluoridated. Despite these limitations, the study sheds light on risk indicators associated with ECC in a rural, socially disadvantaged community in Egypt. Generalization to children in urban settings, with different socioeconomic profile should be done with caution.

## Conclusion

The study identified age, mother’s education, night feeding and frequency of sugary snacks daily, plaque index, and salivary pH as risk indicators for ECC in children in rural communities in Alexandria, Egypt. These factors can aid in targeting at risk children to receive focused preventive regimens and to reduce the burden of ECC and improve children’s oral health.

## Data Availability

The datasets used and analyzed during the current study are available from the corresponding author upon reasonable request.

## Abbreviations

ECC: Early childhood caries
dmft: Decayed, missing and filled teeth for primary dentition
WHO: World Health Organization
